# Continuous Sub-Irrigation with Treated Municipal Wastewater for Protein-Rich Rice Production with Reduced Emissions of CH_4_ and N_2_O

**DOI:** 10.1038/s41598-020-62247-w

**Published:** 2020-03-26

**Authors:** Luc Duc Phung, Dung Viet Pham, Yuka Sasaki, Shuhei Masuda, Fumiaki Takakai, Nobuo Kaku, Toru Watanabe

**Affiliations:** 1 The United Graduate School of Agricultural Sciences, Iwate University, 3-18-8 Ueda, Morioka, Iwate, 020-8550 Japan; 2 Faculty of Agriculture, Yamagata University, 1-23 Wakaba-machi, Tsuruoka, Yamagata, 997-8555 Japan; 3 Department of Civil and Environmental Engineering, National Institute of Technology, Akita College, 1-1 Bunkyo-cho, Iijima, Akita, 011-8555 Japan; 4 Faculty of Bioresource Sciences, Akita Prefectural University, 241-438 Aza Kaidobata-Nishi, Shimoshinjo Nakano, Akita, 010-0195 Japan

**Keywords:** Agroecology, Wetlands ecology

## Abstract

Herein, we introduce continuous sub-irrigation with treated municipal wastewater (TWW) as a novel cultivation system to promote resource recycling and cost-effective forage rice production in Japan. However, both TWW irrigation and forage rice cultivation were previously considered to intensify CH_4_ and N_2_O emissions. In the present study, therefore, we evaluate the emissions of greenhouse gases (GHGs) and yielding capacity of forage rice between conventional cultivation and continuous sub-irrigation systems employing different water supply rates. Results indicated that continuous sub-irrigation with TWW resulted in high rice yields (10.4–11 t ha^−1^) with superior protein content (11.3–12.8%) compared with conventional cultivation (8.6 t ha^−1^ and 9.2%, respectively). All TWW irrigation systems considerably reduced CH_4_ emissions, while higher continuous supply rates significantly increased N_2_O emissions compared with the conventional cultivation. Only the continuous irrigation regime employing suitable supply rates at appropriate timings to meet the N demand of rice plants decreased both CH_4_ and N_2_O emissions by 84% and 28%, respectively. Overall, continuous sub-irrigation with TWW provides high yields of protein-rich forage rice without the need for synthetic fertilisers and effectively mitigated GHG emissions from paddy fields.

## Introduction

Cultivation of forage rice (*Oryza sativa* L.) has been promoted by the Japanese government to reduce the cost of domestic animal husbandry by reducing the use of imported feedstuffs, which can be of unstable supply and highly priced depending on global markets^1^. As a result, there has been a recent increase in forage rice cultivation. However, this has been accompanied by high levels of N fertiliser use to ensure high-yield production, which might lead to inefficient N use and considerable N loss to the environment^2^.

Effluents from wastewater treatment plants (WWTPs) contain high concentrations of organic and inorganic nutrients beneficial for plant growth and development. Thus, reusing these effluents for agricultural irrigation has major advantages for crop production and environmental management^3^. Paddy rice cultivation generally demands large amounts of irrigation water and synthetic fertilisers, and thus, would greatly benefit from recycling water and valuable nutrients from WWTPs. To promote forage rice production and establish an effective resource circulation model for the management of agricultural water, we developed new cultivation systems, and treated municipal wastewater (TWW) was effectively reused in paddy fields to produce high yields of forage rice without applying synthetic fertilisers^1,4,5^.

The reuse of wastewater or TWW for rice cultivation has been intensively investigated and is widely practiced owing to its undeniable benefits. For instance, prior studies have demonstrated that rice grain yield from fields irrigated with treated wastewater could be 35–55% higher than that from groundwater-irrigated fields^6^. Furthermore, reusing wastewater could lower the total cost of rice production by 8.8–11.9%^7^ and reduce ~45% of chemical N fertiliser without jeopardising grain yield^8^. The increase in grain yield and decrease in N fertiliser use could be due to the sufficient supply of nutrients from the wastewater^6,8,9^, which could simultaneously improve soil fertility and increase the metabolic activity of soil microorganisms^3^.

To our knowledge, however, no prior study has thoroughly examined the use of TWW as a sole source of both water and nutrients for paddy rice cultivation, especially for cultivating forage rice, which commonly has higher N demands compared with staple rice varieties^10–12^. In our previous studies, we reported the development of an innovative approach for continuous irrigation with TWW in forage paddy fields, which used TWW as the only source for irrigation and fertilisation^1,4^. In such cultivation systems, TWW was continuously supplied into paddy fields at a constant rate throughout the crop seasons either through an underground drain pipe (sub-irrigation) or on the soil surface (surface irrigation)^1,4^. High grain yields of protein-rich forage rice have been produced using those novel systems, in which higher yield and rice protein content were achieved using the continuous sub-irrigation system compared with the surface irrigation system^4^. As a result, our cultivation systems are expected to promote the low-cost production of protein-rich forage rice and improve the efficiency of farmland, which inevitably secures domestic livestock production and stops the increase in abandoned farmland that has been observed in Japan since the 1970s^13^.

However, forage rice varieties have been recently reported to emit more CH_4_ than common staple rice probably owing to the increased biomass production when cultivated under similar growth conditions, which indicates a possible increase in CH_4_ emissions from the agricultural sector in Japan owing to the increasing production of forage rice^13^. In addition, an earlier study on wastewater irrigation in rice paddy fields claimed that the increased emissions of CH_4_ and N_2_O from wastewater-irrigated fields resulted from the high availability of elements in the wastewater, such as organic C and N^14^. Particularly, paddy fields irrigated with sewage water emitted higher CH_4_ and N_2_O by 27–33% and 68–170%, respectively, compared with conventional fields irrigated with river water^14^. Thus, irrigating forage rice fields with TWW might result in a synergic effect to boost emissions of these greenhouse gases (GHGs) to the atmosphere. Nevertheless, GHG emissions from paddy fields are ultimately influenced by agronomic practices, such as water regime, N fertilisation, and C input^8,12,14,15^. For example, under straw return, TWW irrigation could decrease CH_4_ and N_2_O emissions from paddy fields by 24.5–26.6% and 37–39%, respectively, compared with tap water irrigation^8^. Given the differences in reported GHG emissions from paddy fields under different water and fertilisation practices, our continuous TWW irrigation system requires a thorough investigation to understand its effects on the GHG emissions from forage paddy fields.

Since the continuous irrigation systems use TWW as the sole source for water and fertilisation management, changing irrigation regime would likely lead to substantial shifts in both water and nutrient inputs, which would subsequently influence the emissions of CH_4_ and N_2_O. To estimate the GHG emissions and optimise water regimes of the continuous TWW irrigation systems, we performed a growth chamber experiment to compare CH_4_ and N_2_O emissions from forage paddy fields under continuous sub-irrigation with three different water regimes (R1, R2, and R3) and those under conventional cultivation practices (control). R1 was adapted from our previous study as the preferred irrigation regime^4^, while R2 and R3 were alternatives modified from R1 to test for the optimal regime. The continuous sub-irrigation system was selected for further investigation in the present study owing to its superior agronomic performance compared with the surface irrigation^1,4^. Furthermore, we thoroughly elaborated the differences in grain yield and quality of forage rice under the examined cultivation practices.

## Results

### Grain yield and nutritional quality

**Figure 1:**
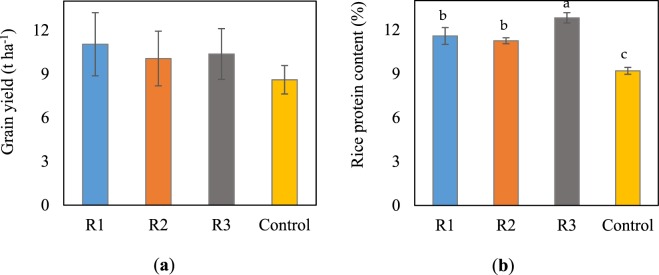
Grain yield (**a**) and rice protein content (**b**) under different cultivation systems. Error bars present standard deviations (n = 4). Different letters indicate significant difference (*p* < 0.05), whereas similar letters or no letter indicate no significant difference.

Although the treatments employing the continuous sub-irrigation systems (R1, R2, and R3) were not supplemented with synthetic fertilisers, their grain yields were not significantly different (*p* > 0.05) from those attained in the control. The lack of fertilisers in R1, R2, and R3 did not decrease the yield but rather tended to increase the yield compared with the application of fertilisers in the control. In particular, the TWW-irrigated treatments produced higher yields (10.1–11 t ha^−1^) than the control (8.6 t ha^−1^) (Fig. 1a), regardless of the water regime used. The yields of R1, R2, and R3 averaged 10.5 t ha^−1^, which was 22% higher than that of the control.

The nutritional quality of rice is generally evaluated based on protein content, which is especially important for feedstuffs. The protein content responded differently to the four cultivation treatments (Fig. 1b). The highest protein content (12.8%) was observed under R3, followed by R1 (11.6%) and R2 (11.3%), while the lowest (9.2%) was observed in the control.

### Heavy metal/metalloid concentrations

Most of the examined elements (except for Cr and Pb) responded differently to the four cultivation treatments (*p* < 0.05), as indicated by the concentrations shown in Table 1. Compared with the control, the TWW-irrigated treatments tended to decrease the concentration of As, Cr, and Cu, but slightly increased the concentration of Cd, Pb, and Zn.

**Table 1:** Concentration of heavy metals/metalloid in the brown rice.

Element (mg kg^−1^)	Treatment				ML^*^	Standard^**^
	R1	R2	R3	Control		
As	0.18 ± 0.01^ab^	0.16 ± 0.03^b^	0.18 ± 0.01^b^	0.21 ± 0.01^a^	0.35	2
Cr	0.04 ± 0.01	0.05 ± 0.02	0.05 ± 0.01	0.06 ± 0.02	NA	NA
Cu	5.01 ± 0.52^bc^	4.61 ± 0.41^c^	5.82 ± 0.26^a^	5.51 ± 0.12^ab^	NA	NA
Cd	0.03 ± 0.01^ab^	0.02 ± 0.01^b^	0.04 ± 0.01^a^	0.02 ± 0.01^b^	0.4	1
Pb	0.07 ± 0.05	0.07 ± 0.02	0.06 ± 0.01	0.06 ± 0.01	0.2	3
Zn	21.7 ± 0.65^ab^	21.9 ± 1.86^a^	23.3 ± 1.58^a^	18.6 ± 0.94^b^	NA	NA

^*^Maximum levels for contaminants and toxin in foods^16^; ^**^Japanese standard for animal feed^17^; NA: not available; Different letters in a row indicate significant difference (*p* < 0.05), whereas similar letters and no letter indicate no significant difference among treatments.

In particular, the concentration of As decreased by 17% under continuous sub-irrigation with TWW. Although As concentration did not differ among R1, R2, and R3 (*p* > 0.05), the lowest As concentration was observed under R2 (0.16 mg kg^−1^). Similarly, TWW irrigation decreased Cr concentration (0.04–0.05 mg kg^−1^) compared with the control (0.06 mg kg^−1^), but this difference was not significant (*p* > 0.05). The same trend was observed for Cu, in which TWW irrigation (R1 and R2) decreased Cu concentration compared with the control, except for a minor increase under R3. The lowest Cu concentration (4.61 mg kg^−1^) was observed under R2 and was 20% lower than that in the control (5.51 mg kg^−1^) (Table 1). The concentration of Cd slightly increased with TWW irrigation under R1 and R3 (0.03 and 0.04 mg kg^−1^, respectively), while R2 maintained the same level as that in the control (0.02 mg kg^−1^). Similarly, Pb concentration increased slightly under R1 and R2, but this increase was not significant (*p* > 0.05), whereas R3 resulted in the same level as the control. TWW irrigation significantly increased Zn concentration (21.7–23.3 mg kg^−1^) compared with the control (18.6 mg kg^−1^) (*p* < 0.05).

Overall, all the examined elements were observed at concentrations lower than the thresholds for contaminants and toxins in foods recommended by FAO/WHO^16^ as well as the Japanese standard for heavy metal/metalloid concentration in animal feed^17^ (Table 1). Among the TWW-irrigated treatments, R2 likely resulted in the lowest accumulation of heavy metals/metalloids.

### Emissions of CH_4_ and N_2_O

**Figure 2:**
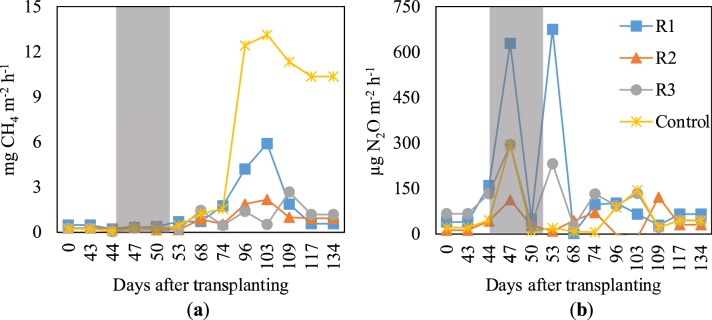
Fluxes of CH_4_ (**a**) and N_2_O emissions (**b**) under different cultivation systems. Gray belts indicate the mid-season drainage (MSD).

**Figure 3:**
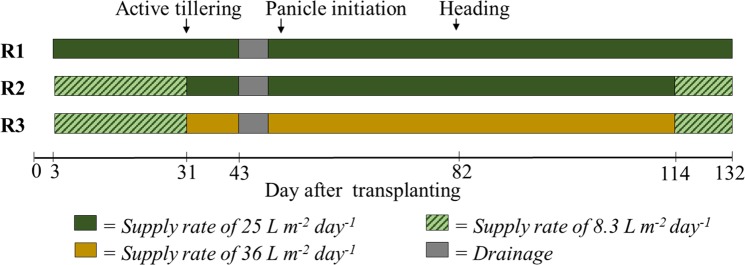
Schematic illustration of the examined water regimes (R1, R2, and R3) under the continuous sub-irrigation systems during the growth period.

Emissions of CH_4_ were not affected by the different cultivation treatments from the beginning of the crop season until the heading stage, around 80 days after transplanting (DAT), whereas substantial fluctuations were observed during the flowering and ripening stages (96 DAT onwards) (Fig. 2a). Before the heading stage, there was no significant difference in CH_4_ flux among the four treatments (0.05–1.76 mg CH_4_ m^−2^ h^−1^). However, the treatments did have a considerable effect as the rice plants started flowering (Fig. 2a). Typically, the control exhibited the highest increase until peaking (13.1 mg CH_4_ m^−2^ h^−1^) at ~103 DAT. Thereafter, CH_4_ emissions gradually decreased at the end of the ripening stage, although at a higher level than those observed under any of the continuous sub-irrigation treatments. A constant supply rate of 25 L m^−2^ day^−1^ throughout the crop season in R1 (Fig. 3) emitted remarkably more CH_4_ than the other water regimes (R2 and R3) under which CH_4_ emissions were likely at the same level (Fig. 2a). The highest seasonal average flux of CH_4_ (4.75 mg CH_4_ m^−2^ h^−1^) was observed in the control, followed by R1 (1.4 mg CH_4_ m^−2^ h^−1^) and R3 (0.79 mg CH_4_ m^−2^ h^−1^), while the lowest was observed under R2 (0.76 mg CH_4_ m^−2^ h^−1^).

Water status was generally divided into two conditions throughout the crop season: a non-waterlogged period during mid-season drainage (MSD) and waterlogging throughout the growth period (Fig. 3). N_2_O emissions were high during the MSD and for ~3 days after (Fig. 2b). Drainage resulted in two high-peak fluxes in R1 (628.4 and 674.4 µg N_2_O m^−2^ h^−1^) and R3 (294.8 and 231.7 µg N_2_O m^−2^ h^−1^), whereas only one peak flux was observed in R2 and the control (112.4 and 293.3 µg N_2_O m^−2^ h^−1^, respectively). Thereafter, fluctuations in N_2_O emissions were observed among the four treatments, regardless of the cultivation practice used. Seasonal fluxes of N_2_O averaged 155.3 and 101.6 µg N_2_O m^−2^ h^−1^ for R1 and R3, which were 163% and 72% higher than that of the control (59.2 µg N_2_O m^−2^ h^−1^), respectively. The lowest average flux was recorded in R2 (38.8 µg N_2_O m^−2^ h^−1^), which emitted 35% less than the control.

Seasonal cumulative emissions of CH_4_ and N_2_O are shown in Table 2. The continuous sub-irrigation systems considerably decreased CH_4_ emissions, regardless of the water regime used. Compared with the control (146.89 kg CH_4_ ha^−1^), continuous sub-irrigation decreased CH_4_ emissions by 70%, 84%, and 83% under R1 (44.77 kg CH_4_ ha^−1^), R2 (24.17 kg CH_4_ ha^−1^), and R3 (25.06 kg CH_4_ ha^−1^), respectively. However, continuous sub-irrigation with TWW increased N_2_O emissions by 170% and 110% for R1 (3.6 kg N_2_O ha^−1^) and R3 (2.81 kg N_2_O ha^−1^), respectively, compared with the control (1.34 kg N_2_O ha^−1^). Conversely, R2 (0.96 kg N_2_O ha^−1^) decreased N_2_O emissions by 28% compared with the control. Overall, although all three continuous irrigation regimes could decrease CH_4_ emissions, R1 and R3 exhibited a trade-off by increasing N_2_O emissions. Only R2 could effectively decrease the emissions of both GHGs.

**Table 2:** Seasonal cumulative emissions of CH_4_ and N_2_O, global warming potentials (GWP), and greenhouse gas intensities (GHGI) under different cultivation systems.

Treatment	Cumulative emissions (kg ha^−1^)		Net GWP (kg CO_2_−eq ha^−1^)	GHGI (kg CO_2_−eq t^−1^)
	CH_4_	N_2_O		
R1	44.77	3.60	2209	206 ± 41^b^
R2	24.17	0.96	932	95 ± 16^c^
R3	25.06	2.81	1446	142 ± 23^bc^
Control	146.89	1.34	4468	525 ± 65^a^

Different letters in a column indicate significant difference (*p* < 0.05), whereas similar letters indicate no significant difference among treatments.

### Net global warming potential and greenhouse gas intensity

Global warming potential (GWP) is an index of the radiative forcing potential of GHGs, with a larger GWP indicating that the gas warms the Earth more efficiently than CO_2_ over a given period of time. Irrespective of irrigation regimes, continuous sub-irrigation considerably decreased net GWP over a 100-year time horizon compared with conventional cultivation (Table 2). The net GWPs of R1, R2, and R3 averaged 1529 ± 643 (kg CO_2_−eq ha^−1^), which was 66% lower than that of the control (4468 kg CO_2_−eq ha^−1^). This result suggests that continuous sub-irrigation with TWW would substantially diminish the radiative forcing of forage rice paddy fields. The modified regimes R2 and R3 reduced the net GWP of R1 by 58% and 35%, respectively. Overall, the lowest net GWP (932 kg CO_2_−eq ha^−1^) among the four examined treatments was observed in R2 (Table 2).

Greenhouse gas intensity (GHGI) is an index commonly used to represent the efficiency of rice production systems by linking grain yield with the corresponding net GWPs, for which the effects of producing a certain grain yield on the climate during cultivation is estimated. Since no significant difference was observed among grain yields (Fig. 1a), the GHGIs of the four treatments followed the same trend observed in the net GWP, in which the highest (525 ± 65 kg CO_2_−eq t^−1^) was recorded in the control and the lowest (95 ± 16 kg CO_2_−eq t^−1^) in R2.

## Discussion

**Figure 4:**
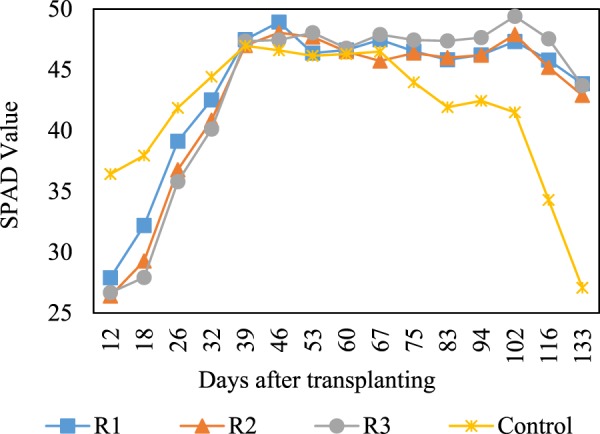
Leaf greenness (SPAD value) of the rice plants during the growing period.

The higher grain yields achieved in the TWW-irrigated systems were probably attributed to the continuous supply of plant nutrients, especially N, contained in the TWW at high concentrations during the growing season (Table 3). Being the most yield-limiting nutrient in rice production, N is generally applied through high doses of synthetic fertilisers to ensure high levels of rice production, especially for high-yielding cultivars such as the forage rice ‘Bekoaoba’ used in this study^12,15^. In this experiment, the continuous sub-irrigation systems supplied large amounts of N into R1, R2, and R3 (~811, 575, and 778 kg N ha^−1^, respectively), which were 3.1, 2.2, and 3.0-fold higher than that supplemented by the fertilisers in the control (260 kg N ha^−1^). As a result, the TWW-irrigated plants maintained considerably higher leaf greenness, which was measured using a chlorophyll meter and expressed as SPAD values^4^, compared with the rice under conventional cultivation during the flowering and grain filling stages (~80 DAT onwards, Fig. 4).

**Table 3:** Basic properties of the irrigation TWW during the growth period.

Parameters	Unit	May	Jun	Jul	Aug	Sep	Mean
pH		7.5	7.2	7.5	7.4	7.0	7.3
EC	mS m^−1^	70	63	68	61	54	63
DO	mg L^−1^	4.7	4.3	3.2	3.4	4.6	4.0
TOC	mg L^−1^	6.2	5.8	6.0	5.5	5.4	5.8
TN	mg L^−1^	37	33	27	22	21	28
TP	mg L^−1^	0.7	0.5	0.4	0.6	0.9	0.6
K	mg L^−1^	9.2	9.5	11.0	9.5	9.2	9.7
As	µg L^−1^	0.2	0.4	0.2	0.4	0.5	0.3
Cr	µg L^−1^	1.0	0.6	0.9	0.5	0.6	0.7
Cu	µg L^−1^	9.4	8.7	8.5	7.9	8.1	8.5
Cd	µg L^−1^	<0.1	<0.1	<0.1	<0.1	<0.1	<0.1
Pb	µg L^−1^	5.4	4.2	2.9	1.3	0.6	2.9
Zn	µg L^−1^	42	49	56	50	30	45

Since the SPAD value is strongly associated with N status in leaves^18^, it is one of the best indicators of photosynthetic activities in rice. Generally, starch and sugar accumulate at high levels in rice culms and leaf sheaths before flowering, and the accumulated carbohydrates are translocated into rice grains during the grain filling stage, which causes the rice culms and leaves to senesce and turn yellowish^19^. By delaying the senescence and maintaining effective photosynthesis, more carbohydrates could be produced and be available for grain filling, subsequently resulting in higher grain yields. Our results suggest that the N derived from TWW could sufficiently substitute for the N supplemented by synthetic fertilisers. These results are consistent with those of a previous study^20^, which demonstrated that N-rich wastewater was as effective as commercial N fertiliser at achieving optimum rice yields. This was further supported by our previous study, which reported that continuous sub-irrigation removed 85–90% of the N available in TWW^4^, and the utilisation of newly absorbed N until the late growth period is critical for producing the high yields of high-yielding rice varieties^21^.

Water regime R1 was the preferred water scheme demonstrated in our previous study^4^; however, continuous irrigation at a constant rate of 25 L m^−2^ day^−1^ throughout the growing season probably reduced the efficient use of N owing to the variable N demand of rice plants in each growth stage^19^. In the present study, we modified this water regime into R2 and R3 to test for an optimal regime. The lower supply rate of 8.3 L m^−2^ day^−1^ was used during the early growth stage and near maturity when the rice plants generally had a low N-absorbing capacity^19^. A higher supply rate of either 25 L m^−2^ day^−1^ or 36 L m^−2^ day^−1^ was used in R2 or R3, respectively, from 31 to 114 DAT, which was a period of high N demand, whereas active tillering, panicle initiation, heading, and grain filling occurred consecutively (Fig. 3) with a high N-use efficiency to maximise tiller number, increase panicle size, improve filled-grain percentage, and enhance grain weight^19^. Compared with R1, R2 and R3 did not cause significant yield losses (*p* > 0.05, Fig. 1a), suggesting an opportunity to apply suitable supply rates at appropriate timings to meet the N demands of the rice plants. The better N assimilation during the late growth period also explained the increased rice protein content observed in R1, R2, and R3 (Fig. 1b). The highest protein content obtained in R3 (*p* < 0.05) can be attributed to the higher supply rate of 36 L m^−2^ day^−1^ maintaining the most efficient photosynthesis in the rice plants from 67 DAT onwards (Fig. 4).

Overall, our study provides evidence to suggest that continuous sub-irrigation with TWW is an effective means to reuse the effluents from WWTPs to produce high yields with high protein content without using exogenous synthetic fertilisers. This result agreed with those of previous studies^1,4,9^, suggesting a cost-effective strategy for recycling water and plant nutrients that simultaneously reduces the demand for synthetic fertilisers and the amount of nutrients discharged into surface water bodies. Furthermore, eliminating the use of fertilisers will not only decrease the adverse environmental effects but will also increase profits for farmers^7,9^. Relative to the constant supply rate in R1, combining more suitable supply rates with relevant timings in R2 and R3 could maintain the high yielding capacity and high rice protein content (Fig. 1).

Accumulation of heavy metals/metalloids by crops irrigated with wastewater has generally been considered an environmental problem since those metals tend to accumulate in soil and could become bioavailable for crops^3^. However, in the present study, there was no notable adverse effect of TWW irrigation on the accumulation of toxic elements, like As, Cr, Cu, Cd, and Pb, in the rice grains. This result is in accordance with those of other studies^5,6,9^, which reported that the chemical compositions of rice irrigated with TWW were within the common range observed in conventional paddy fields. The slight increase in Zn content in the rice grains under TWW irrigation compared with that under the control (Table 1) was probably due to the higher concentration of Zn in TWW relative to other elements (Table 3). Interestingly, Zn is one of the most essential micronutrients for humans, and thus, attempts have been made to improve Zn content in rice^22^. The lower concentrations of As, Cr, and Cu observed under continuous sub-irrigation with TWW relative to the control were probably attributed to the continuous overflow of water that might carry these elements out of the paddy soils. Ultimately, although minor variation in the concentrations of the examined elements was observed among the continuous sub-irrigation water regimes (R1, R2, and R3), and between those and the control, all the concentrations were lower than the maximum limits recommended by FAO/WHO^16^ and the Japanese standard^17^ (Table 1), suggesting that the rice grains harvested from the continuous sub-irrigation systems are safe to use as feedstuffs.

The production of CH_4_ in rice fields generally results from the anaerobic decomposition of organic matter in rhizosphere soil. In contrast with a previous study^14^, which reported an increase in CH_4_ emissions from paddy fields irrigated with wastewater, continuous sub-irrigation considerably decreased the seasonal emissions of CH_4_ by 70–84% compared with conventional cultivation (Table 2). This decrease was probably due to the substantial amounts of dissolved oxygen maintained in the TWW (Table 3) being continuously supplied into the rhizosphere as TWW was pumped into the deep soil layers, which might subsequently inhibit methanogen communities and their activities regarding CH_4_ production. The high peaks of CH_4_ fluxes recorded in all treatments during the grain filling stage were mainly attributed to the higher availability of C substances in the paddy soil as a result of enhanced root exudation during flowering. The highest exudation rates were observed during the grain filling stage compared to the other growth stages^23^. Since root exudates provide C substrates for methanogenesis in rice fields, the higher root exudation during flowering could greatly stimulate CH_4_ emissions in the following grain filling stage^23^. Among the three continuous irrigation regimes tested in the present study, R2 was the most effective in terms of CH_4_ mitigation (Fig. 2, Table 2), probably owing to the lowest input amount of available C accompanied by the lowest irrigation rates (Fig. 3).

The emissions of N_2_O in the control, which contributed to the net GWP by 8%, were low compared with CH_4_ emissions. This result agrees with those of other studies that reported negligible N_2_O emissions in flooded paddy fields under conventional cultivation^24,25^. The high peaks of N_2_O fluxes observed in all treatments during MSD were consistent with the common phenomenon in paddy fields under field drainage that promotes N_2_O emissions due to enhanced nitrification-denitrification processes under favourable conditions^14,15^. The additional peaks recorded in R1 and R3 within 3 days afterwards were likely due to the high N concentration in the soil and surface water when TWW was re-supplied into the experimental chambers. Emissions of N_2_O from R1, R2, and R3 contributed to 43%, 27%, and 51% of the net GWP, respectively, probably due to the high N concentration in the TWW continuously supplied into the chambers (Table 3). The lowest emission of N_2_O recorded in R2 relative to the other TWW-irrigated treatments (Table 2) was mainly attributed to the lowest N input accompanied by lower irrigation rates. It is likely that the lower fertilisation in R2 ensured the efficient use of nutrients by the rice plants, leaving very little residual N for nitrification and denitrification, thereby reducing N_2_O emissions. The higher N_2_O emissions from R1 and R3 were essentially due to the enhanced nitrification/denitrification process induced by the considerable N contained in the TWW and the higher supply rates (Fig. 3). Furthermore, the rich sources of organic matter supplied by TWW could also benefit N-cycling bacterial communities^14^, subsequently increasing N_2_O emissions. Overall, our results indicate that R2 is the most effective mitigator that can overcome the trade-off between N_2_O and CH_4_ emissions compared with R1 and R3.

Efficient cultivation practices must involve producing the optimum rice yield along with low environmental effects. Prior studies have reported many potential practices to increase rice yield and simultaneously mitigate GHG emissions from paddy fields^26,27^. In the present study, continuous TWW irrigation considerably decreased net GWPs primarily owing to the considerable decrease in CH_4_ emissions (Table 2). The combination of two supply rates in R2 and R3 (Fig. 3) tended to decrease seasonal CH_4_ and N_2_O emissions (Fig. 2), and subsequently diminish the net GWPs compared with the constant supply rate in R1. The lowest net GWP and GHGI attained in R2 is attributed to its most effective minimisation of both CH_4_ and N_2_O emissions. Our results have shown that appropriately matching the lower (8.3 L m^−2^ day^−1^) and higher (25 L m^−2^ day^−1^) supply rates with the periods of low and high N demand of rice plants (Fig. 3), respectively, leads to R2 being the optimised irrigation regime for continuous sub-irrigation with TWW to reduce the GHG budget of rice paddy fields without significant yield loss or protein reduction.

In conclusion, the continuous sub-irrigation systems produce high yields of protein-rich forage rice through the reuse of TWW as the sole source for both irrigation and fertilisation in paddy fields. Importantly, by employing the optimal water regime (R2), continuous sub-irrigation with TWW can effectively mitigate CH_4_ and N_2_O emissions from the rice paddies. The practice of recycling valuable plant nutrients contained in TWW to meet the high nutrient demand of forage rice production instead of applying high doses of synthetic fertilisers demonstrates the potential to minimise the dependence of rice production on fertilisers, which would simultaneously mitigate GHG emissions and promote sustainable rice paddy farming. Our results will motivate local farmers to adopt the continuous irrigation systems to reuse TWW for rice cultivation. The agronomic performance and cost efficiency of adopting these practices under real farm conditions will be analysed by our research team in future studies.

## Methods

### Experimental design

**Figure 5:**
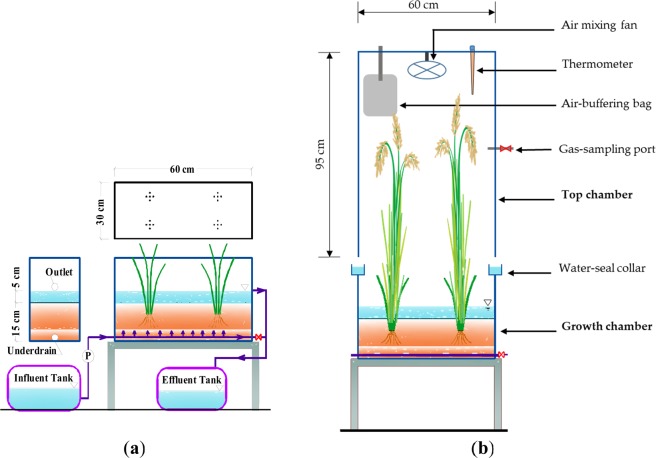
Schematic illustration of the growth chamber employing the continuous sub-irrigation systems (**a**) and the top chamber used for sampling gases (**b**).

The experiment was conducted in the 2018 crop season at Yamagata University, Tsuruoka City, Japan, using four polyethylene growth chambers (36 cm height, 30 cm width, 60 cm length) to simulate paddy fields of 0.18 m^2^ (Fig. 5a). At the bottom of each chamber, a perforated drainage pipe (21 cm diameter) was installed and covered by a layer of gravel. The chambers were filled with 30 kg of a loamy soil (air-dried, 20% moisture) with a pH of 5.5, an EC of 9.3 mS m^−1^, and total C, N, P, and K content of 20000, 1710, 920, and 1839 mg kg^−1^, respectively. In each chamber, four hills of forage rice ‘Bekoaoba’ were transplanted at a plant density of 15 × 30 cm (Fig. 5a).

Four treatments were used in the present study, including one under conventional farming practices (control) and three under continuous sub-irrigation with TWW each with a different water regime (Fig. 3). TWW used for the continuous irrigation was collected from a local WWTP and monitored weekly for its physicochemical properties following the standard methods described in our previous study^5^. The basic properties of the irrigation TWW were shown in Table 3.

The control treatment was fertilised with 160 kg N-P_2_O_5_-K_2_O ha^−1^ as basal at 1 day before transplanting and 100 kg N-K_2_O ha^−1^ at the panicle–initiation stage. The fertiliser dose was adjusted based on the chamber’s surface area basis, while daily watering involved manually adding tap-water to maintain 5-cm standing water above the soil surface. The treatments employing continuous sub-irrigation systems were setup by pumping TWW stored in an influent tank into the chambers through the perforated pipes, from which TWW infiltrated the soil layer and ultimately overflowed into effluent tanks via the outlets installed at 5 cm above the soil surface (Fig. 5a). Three water regimes of continuous sub-irrigation were controlled by electric pumps (EYELA Cassette Tube Pump SMP-23), set to a constant supply rate of 25 L m^−2^ day^−1^ throughout the crop season (R1); a supply rate of either 25 (R2) or 36 L m^−2^ day^−1^ (R3) from 31 to 114 DAT combined with a lower rate of 8.3 L m^−2^ day^−1^ for the other growing periods (Fig. 3). Irrigation with TWW was initiated at 3 DAT and maintained continuously throughout the crop season, except for 1 week of MSD that is conventionally practiced locally (Fig. 3). The treatments employing three continuous sub-irrigation regimes were not supplemented with exogenous fertilisers; thus, TWW was the only source of water and fertilisation inputs. Total N inputs of R1, R2, and R3 were approximately 811, 575, and 778 kg N ha^−1^, respectively, which were theoretically estimated based on the supply rates and the N concentration in the TWW during the crop season.

### Measurement of grain yield and rice quality

Grain yield was measured at harvesting and presented as the weight of brown rice after being adjusted to 14% moisture content. The nutritional quality of the harvested grains was evaluated based on rice protein content, which was calculated by multiplying a conversion factor of 5.95^28^ by the total N content of the brown rice analysed by an automatic high-sensitivity NC analyser (Sumigraph NC-220F, SCAS, Japan). Concentrations of heavy metals/metalloids (As, Cr, Cu, Cd, Pb, and Zn) in the brown rice were determined by the standard wet-digestion method followed by measuring with either an atomic absorption spectrometer (AAS Model AA7000 equipped with hydride generator, Shimadzu Corporation, Japan) for As, or an inductively coupled plasma mass spectrometer (ICP-MS) (Elan DRC II, PerkinElmer, Japan) for the other elements^29^.

### Gas sampling and analysis

Gas samples of CH_4_ and N_2_O were collected during the growing season (May to September 2018) following the manually operated closed chamber method^30^. We used transparent compartments (top chambers) (95 cm height, 30 cm width, 60 cm length) that could be mounted on the growth chambers on water-seal collars for gas-sampling events (Fig. 5b). Each compartment was equipped with one battery-driven fan to homogenise the air inside, an air-buffer 2-L plastic bag to compensate for pressure changes, a thermometer to monitor temperature changes during the gas-sampling period, and a gas-sampling port equipped with a 3-way stopcock valve for collecting gas samples from the compartment (Fig. 5b).

Gas samples was collected weekly from 10.00 to 11.00 am. A 50-mL plastic syringe equipped with a 3-way stopcock valve was used to collect gas samples from the top chambers at 0, 15, and 30 min after deployment. The collected gas samples were immediately transferred to 10-mL air-evacuated glass vials and transported to the laboratory for measuring CH_4_ and N_2_O concentrations using a gas chromatograph (Shimadzu GC-2014, Kyoto, Japan). Fluxes and seasonal cumulative emissions of CH_4_ and N_2_O were estimated following standard procedures^30^. Briefly, CH_4_ and N_2_O fluxes were calculated by examining the linear increases in CH_4_ and N_2_O concentrations in the top chambers over time. The seasonal cumulative CH_4_ and N_2_O emissions from all chambers were calculated directly from the fluxes. To estimate the net GWP of each treatment, the seasonal cumulative CH_4_ and N_2_O emissions were converted to CO_2_−equivalents (CO_2_−eq) using a radiative forcing potential relative to CO_2_ over a 100-year time horizon of 28 for CH_4_ and 265 for N_2_O^31^. The GHGI was calculated by dividing the net GWP by the grain yield^27^.

### Statistical analysis

The four rice plants transplanted into each treatment chamber were treated as four replicates to analyse the data pertaining to grain yield, rice protein content, and concentration of heavy metals/metalloids. Data were subjected to analysis of variance (ANOVA), and the means of significant treatment effects were compared using Tukey’s honestly significant difference test (HSD) at the 5% probability level using IBM SPSS Statistics 24.0. Differences in CH_4_ and N_2_O emissions among four treatments were examined based on a single data point basis.
